# The Potential of PIP3 in Enhancing Wound Healing

**DOI:** 10.3390/ijms25031780

**Published:** 2024-02-01

**Authors:** Yossi Blitsman, Etili Hollander, Chen Benafsha, Ksenia M. Yegodayev, Uzi Hadad, Riki Goldbart, Tamar Traitel, Assaf Rudich, Moshe Elkabets, Joseph Kost

**Affiliations:** 1 Department of Chemical Engineering, Ben-Gurion University of the Negev, Beer-Sheva 8410501, Israel; blitsman@post.bgu.ac.il (Y.B.); chenbere@post.bgu.ac.il (C.B.); goldbart@post.bgu.ac.il (R.G.); tamluz@post.bgu.ac.il (T.T.); 2 The Shraga Segal Department of Microbiology, Immunology and Genetics, Faculty of Health Sciences, Ben-Gurion University of the Negev, Beer-Sheva 8410501, Israel; kseniay@post.bgu.ac.il (K.M.Y.); moshee@bgu.ac.il (M.E.); 3 The Ilse Katz Institute for Nanoscale Science and Technology, Marcus Campus, Ben-Gurion University of the Negev, Beer-Sheva 8410501, Israel; uzihad@bgu.ac.il; 4 Department of Clinical Biochemistry and Pharmacology, Faculty of Health Sciences, Ben-Gurion University of the Negev, Beer-Sheva 8410501, Israel; rudich@bgu.ac.il

**Keywords:** phosphatidylinositol 3,4,5-trisphosphate (PIP3), wound healing, intracellular delivery, cationic starch (Q-starch), drug delivery

## Abstract

Given the role of phosphatidylinositol 3,4,5-trisphosphate (PIP3) in modulating cellular processes such as proliferation, survival, and migration, we hypothesized its potential as a novel therapeutic agent for wound closure enhancement. In this study, PIP3 was examined in its free form or as a complex with cationic starch (Q-starch) as a carrier. The intracellular bioactivity and localization of free PIP3 and the Q-starch/PIP3 complexes were examined. Our results present the capability of Q-starch to form complexes with PIP3, facilitate its cellular membrane internalization, and activate intracellular paths leading to enhanced wound healing. Both free PIP3 and Q-starch/PIP3 complexes enhanced monolayer gap closure in scratch assays and induced amplified collagen production within HaCAT and BJ fibroblast cells. Western blot presented enhanced AKT activation by free or complexed PIP3 in BJ fibroblasts in which endogenous PIP3 production was pharmacologically inhibited. Furthermore, both free PIP3 and Q-starch/PIP3 complexes expedited wound closure in mice, after single or daily dermal injections into the wound margins. Free PIP3 and the Q-starch/PIP3 complexes inherently activated the AKT signaling pathway, which is responsible for crucial wound healing processes such as migration; this was also observed in wound assays in mice. PIP3 was identified as a promising molecule for enhancing wound healing, and its ability to circumvent PI3K inhibition suggests possible implications for chronic wound healing.

## 1. Introduction

A wound is characterized as an interruption in the structural integrity of any body tissue due to external factors, which can range from surgical procedures to other external forces [1]. Acute wound healing, spanning roughly 12 weeks, can be categorized into four stages. It begins with hemostasis when blood vessels constrict and form clots to stop bleeding. Following this, inflammation sets in, characterized by the body’s immune response clearing debris and preventing infections at the wound site. Subsequently, during the proliferation stage, new tissue starts forming, including the production of collagen, essential for wound closure and contraction. Lastly, in the remodeling phase, the newly formed tissue matures and strengthens as collagen realigns, enhancing the overall integrity and functionality of the healed area [2,3,4,5,6]. A chronic wound arises when healing is prolonged for more than three months, which is attributed to disruptions in any of the recovery stages [7,8,9,10]. Predominantly, venous and arterial ulcers, diabetic ulcers, and pressure ulcers represent the bulk of chronic wounds [11,12,13]. As for 2020, global chronic wound prevalence was approximated at 6.7 million individuals. Concurrently, the chronic wound care market reached a valuation of USD 11.61 billion in 2021 and is anticipated to ascend to USD 19.52 billion by 2027 [14]. Numerous advanced dressings cater to the optimal cellular environment, moisture management, gas interchange, and inflammation mitigation [15,16,17,18]. Yet, a definitive solution for chronic wounds remains elusive.

The AKT/mTOR pathway is essential for cell migration, and its compromised function hinders processes like cellular proliferation and wound healing [19,20]. In the context of acute wound healing processes, there is an observed gradual increase in phosphorylated Akt (p-Akt), specifically at the boundary of the wound, in comparison to the intact skin surrounding the wound site. Research findings indicate that the total level of Akt protein showed notable elevation throughout all stages after injury in non-diabetic rats’ wounds. In contrast, this increase was absent in wounds of diabetic rats (with diabetes induced by streptozotocin) compared to the intact skin (day 0) of non-diabetic and diabetic rats [21]. Moreover, the levels of phosphorylated Akt (p-Akt) and phosphorylated mTOR proteins in both non-diabetic and diabetic wounds displayed an upward trend from day 1 to day 7 post wounding. However, it is noteworthy that the levels of these phosphorylated proteins in diabetic wounds were consistently lower than those observed in non-diabetic wounds at corresponding time points [21].

To overcome this AKT/mTOR downregulation in diabetic wounds, there are several biological treatments known today. Bioengineered skin substitutes, like Apligraf or Dermagraft, utilize a skin substitute comprising living cells integrated into a matrix that provides structural support. The graft is positioned at the site of the chronic wound, facilitating the process of wound healing and recovery [22,23,24]. Another treatment, the sole biologically based treatment that is based on the activation of the AKT cascade, approved by the FDA, employs platelet-derived growth factor and is named Becaplarmin [25,26]. The complete closure for ulcers, with this treatment and good ulcer care for 20 weeks or until the target ulcer was completely healed, was approximately 50%, compared to 35% with a placebo gel and good ulcer care, and decreased the time to achieve complete wound closure from 127 days to 86 days [27]. Despite the range of available wound treatments, an optimal solution with consistently high rates of wound closure has yet to be identified.

Phosphatidylinositol (PtdIns) serves as a fundamental lipid constituent of cellular membranes, playing a pivotal role in critical metabolic pathways across both plant and animal kingdoms [28,29,30,31,32]. Various lipid kinases can phosphorylate PtdIns, targeting the positions 3, 4, and 5 of the inositol ring, forming seven distinct PtdIns species [33,34], in particular PIP3, which is an essential second messenger. PIP3 is involved in engaging and activating an array of regulatory proteins at the plasma membrane, thereby influencing cell morphology, motility, and an extensive range of additional cellular processes through the AKT signaling pathway [35,36,37,38,39,40].

For wound healing to occur, the neighboring cells must endure, multiply, migrate, and grow directionally to seal the wound. This can be achieved by activating the AKT/mTOR signaling cascade, which is activated by receptor tyrosine kinases, integrins, and other stimuli that induce the production of PIP3 through the phosphorylation of phosphatidylinositol 4,5-bisphosphate (PIP2) by phosphoinositide 3-kinase (PI3K) [36,39]. In light of this, we propose an innovative therapeutic strategy consisting in the incorporation of exogenous PIP3 to trigger intracellular functions during the proliferative phase of wound healing, enhancing cell proliferation and migration, and facilitate wound closure by directly stimulating AKT. The introduction of PIP3 can circumvent upstream processes that activate PI3K for endogenous PIP3 production. Furthermore, activating intracellular metabolic processes can potentially counteract disruptions in wound healing resulting from infections, ischemia, or conditions like diabetes.

Throughout this research, evaluations of PIP3 in cells and in mice were conducted on both free PIP and PIP within a complex, assessing the system’s resilience to potential future challenges. Furthermore, employing a carrier would facilitate the prospective utilization of PIP3 as a controlled-release therapy. To address this, a modified polysaccharide carrier was used.

In this study, we employed a cationic polysaccharide derived from potato starch, embedded with quaternary amine groups, to serve as a PIP3 carrier. This Q-starch carrier spontaneously formed nanoparticles via ionic interactions with the PIP3 molecule. These complexes were evaluated in vitro using HaCAT keratinocyte and BJ fibroblast cell lines as representatives for different types of skin cells. During the cellular uptake trials, the Q-starch/PIP3 complexes were localized within the cell membrane, the endogenous site of PIP3 activity, and demonstrated biological efficacy. Cells exposed to both free and complexed PIP3 exhibited AKT activation, enhanced proliferation, accelerated migration, and increased collagen synthesis, which are crucial components of the wound healing process. Notably, the wounds exhibited accelerated healing in the initial days after a single application of free of PIP3 and the Q-starch/PIP3 complexes, highlighting the PIP3 potential as a viable therapeutic intervention for wound repair.

## 2. Results and Discussion

### 2.1. Quaternized Starch Chemical Analysis

Starch modification is essential for the addition of a positive charge to the neutral polymer backbone. This enables the self-assembly process with the negatively charged PIP3, to form complexes. The chemical modification was conducted by substituting one of the hydroxyl groups on the polymer backbone with a quaternary amine group (Q-starch), as described in our previous studies [41,42,43]. Q-starch synthesis was characterized using 1H NMR, elemental analysis, and FT-IR spectroscopy, which presented the successful introduction of the quaternary groups on the starch backbone [43].

### 2.2. Q-Starch/PIP3 Complexes Characterization

The ratio enabling complete complexation and triggering a shift from a negative to a positive zeta potential was determined by the characterization of the Q-starch/PIP3 complexes at different N/P ratios, where (N) represents the molar fraction of positive nitrogen groups on the carrier, and (P) represents the molar fraction of negative phosphate groups on PIP3. The dimension and morphology of the Q-starch/PIP3 complexes were examined using dynamic light scattering (DLS), cryogenic transmission electron microscopy (cryo-TEM), and atomic force microscopy (AFM).

**Figure 1:**
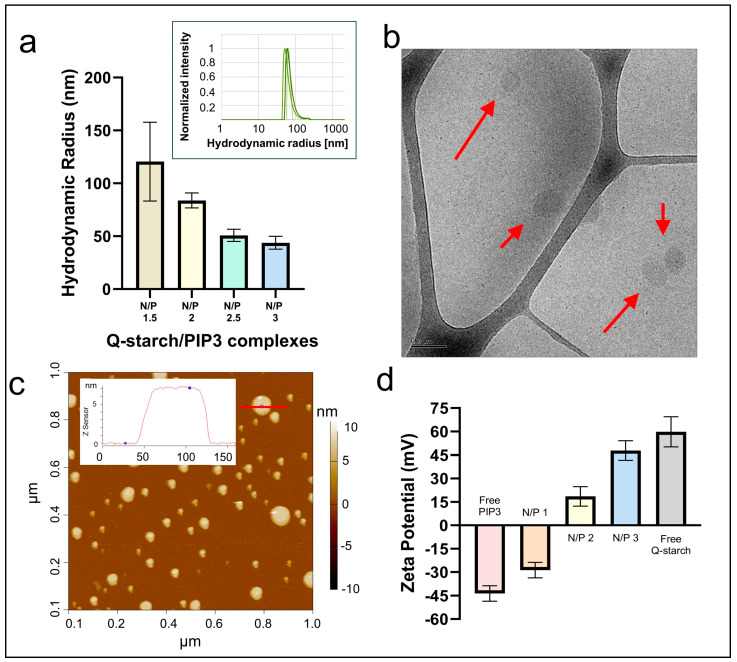
Characterization of the Q-starch/PIP3 complexes. (**a**) DLS of the Q-starch/PIP3 complexes at different N/P ratios; PIP3 concentration was 250 nM. Upper square: size distribution of the Q-starch/PIP3 complexes at a N/P ratio of 2 from 3 independent experiments. (**b**) Cryo-TEM of Q-starch/PIP3 at a N/P ratio of 2; PIP3 concentration was 250 nM, red arrows point to the Q-starch/PIP3 complexes. (**c**) AFM of the Q-starch/PIP3 complexes at a N/P ratio of 2; PIP3 concentration was 250 nM. The inset presents an analysis of one of the particles as an example. (**d**) Zeta potential of Q-starch/PIP3 complexes at different N/P ratios; PIP3 concentration was 50 nM, free Q-starch concentration corresponding to that in the Q-starch/PIP3 complexes at a N/P ratio of 2.

The complexation and the size of the resulting Q-starch/PIP3 complexes were measured using DLS, as shown in Figure 1a. At a N/P ratio of 1.5, complexes with a mean radius of 121 nm were measured. At a N/P ratio of 2, the complexes’ size was about 84 nm and continued to decrease with the increase in the N/P ratio up to a size of 44 nm at the N/P ratio of 3. The obtained complexes had a narrow size distribution; a representative example can be seen in Figure 1a inset, in which complexes are presented for a N/P ratio of 2.

The cryo-TEM and AFM characterizations of complexes at a N/P ratio of 2 (Figure 1b,c) revealed that these complexes possessed a spherical configuration. Spherical complexes can offer advantages in terms of stability, efficient delivery, or interactions with biological systems due to their compact and often uniform shape [44,45].

Regarding the zeta potential, as presented in Figure 1d, free PIP3 exhibits a negative charge, whereas the free carrier, Q-starch, displays a positive one. When the carrier and PIP3 were complexed, an increase in the positive charge with the increase in the N/P ratio could be observed. A transition from negative to positive zeta potential was observed at a N/P ratio of 2, and at a N/P ratio of 3, an additional increase in the zeta potential was observed.

For successful cellular uptake, the literature suggests that complexes need to be positively charged, which counteracts the electrostatic repulsion of the negatively charged cell membrane [46]. Here, by using Q-starch as a carrier, it was possible to overcome the repulsion of the negative zeta potential of PIP3 and the cell membrane. The literature suggests that particles with zeta potentials ranging from below −30 mV to +30 mV, which was the zeta potential value range of our complexes, may tend to aggregate [47]. However, in this study, no aggregation was observed across all conducted experiments, as indicated by the DLS results, showing a narrow size distribution (Figure 1a). We assumed that the absence of aggregation was observed due to sterical stabilization. The bond that creates the complex occurs between the PIP3 inositol head, which consists of three negative sites, and a positively charged quaternary amine. In addition, PIP3 consists of two hydrophobic chains that may cause electrostatic hindrance. This balance of electrostatic forces potentially maintains the equilibrium between particle repulsion and attraction, hindering particle aggregation [48].

All the Q-starch/PIP3 complexes for the DLS and the zeta potential measurements were prepared at a PIP3 concentration of 500 nm. Due to equipment limitations, the examined Q-starch/PIP3 complex solutions were diluted right before the measurements, reaching, for the DLS measurement, a final concentration of 250 nM and, for the zeta potential measurements, a final concentration of 50 nM. Usually, higher concentrations raise the possibility of aggregation. Since no aggregation was observed in the DLS measurements, we assumed that no aggregation occurred in the zeta potential measurements, when the concentration was lower.

In order to maintain the biological activity of PIP3 and the possibility of its release from the complexes, a relatively low zeta potential was needed. Based on the indicated characterizations, the shift in zeta potential from negative to positive, and the globular homogenous nanometric size, we decided to proceed with experiments involving complexes with a N/P ratio of 2.

### 2.3. Cellular Uptake of the Q-Starch/PIP3 Complexes

**Figure 2:**
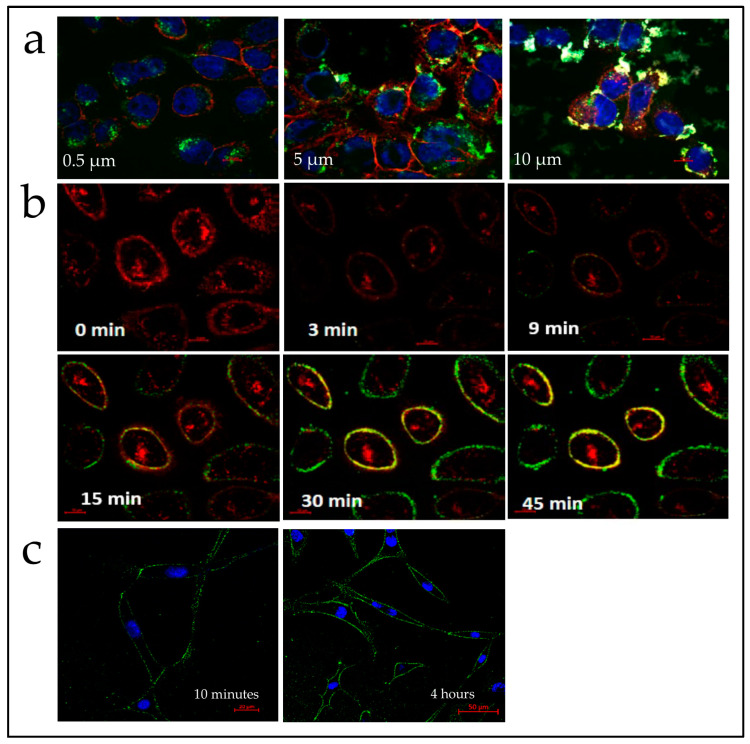
(**a**) Live confocal images of Q-starch^DTAF^/PIP3 complexes’ cellular uptake at different PIP3 concentrations after 30 min in HaCAT cells. Bar—10 µm. Blue—cell nuclei, red—membrane, green—Q-starch^DTAF^/PIP3 complexes. (**b**) Live confocal images of Q-starch^DTAF^/PIP3 complexes’ cellular uptake in HaCAT cells. Numbers indicate the time passed since complex introduction. PIP3 concentration, 0.5 µM. Bar—10 µm. Red—membrane, green—Q-starch^DTAF^/PIP3 complexes. (**c**) Confocal images of Q-starch^DTAF^/PIP3 complexes’ cellular uptake in fibroblast cells. Numbers indicate the time passed since complex introduction. PIP3 concentration, 0.5 µM. Bar (**left**)—20 µm, bar (**right**)—50 µm. Blue—cell nuclei, green—Qstarch^DTAF^/PIP3 complexes.

Endogenous PIP3 derives from PIP2 phosphorylation by PI3K on the cell membrane, triggering subsequent intracellular activities [49,50]. To observe the activity of PIP3 within the Q-starch/PIP3 complexes, it was crucial to note an accumulation of the complexes on the cell membrane. To visualize the localization of the complexes, the Q-starch carrier was labeled with DTAF5, enabling its observation using confocal microscopy. The optimal concentration for cellular uptake and biological activity of the Q-starch/PIP3 complexes was derived after evaluating Q-starch/PIP3 complexes at the N/P ratio of 2 and PIP3 concentrations between 500 nM and 10 µM. As can be seen in Figure 2a, Q-starch^DTAF^/PIP3 complexes at the PIP3 concentration of 500 nM accumulated mostly in the cell membrane of HaCAT cells after 30 min. At higher concentrations of 5 and 10 µM, a very high confluency was observed, with infiltration of the complexes into the cytoplasm. As shown in Figure 2b, time-lapsed confocal images were taken to observe the Q-starch^DTAF^/PIP3 complex cellular uptake over 45 min. Remarkably, within just 9 min, the Q-starch^DTAF5^/PIP3 complexes were visible on the HaCAT cells’ membrane, where they persisted throughout the 45 min of observation, showing no signs of cytoplasmic infiltration.

In addition to HaCAT cells, the complex cellular internalization was assessed in skin-derived BJ fibroblast cells, which are also pivotal in wound healing processes. As seen in HaCAT cells, Q-starch^DTAF^/PIP3 complexes were visible on the BJ fibroblast cell membrane (Figure 2c) as early as 10 min after their introduction. In the images taken after 4 h, the complexes persisted prominently on the cell membrane, comparable to what observed in HaCAT cells, suggestive of their potential for prolonged activity.

These results suggest Q-starch^DTAF^/PIP3 complexes’ potential for targeted action on the cellular membrane where endogenous PIP3 is located and active. For all further experiments, Q-starch/PIP3 complexes with a PIP3 concentration of 500 nM and a N/P ratio of 2 were chosen.

The outcomes of this experiment are similar to those of an earlier study employing PEI25 as a carrier for PIP3 [51]. Polyethyleneimine (PEI-25, 25 kDa) was previously identified as an effective vehicle for PIP3, forming complexes observed on the cell membrane within approximately one minute. Conversely, the Q-starch/PIP3 complexes took about 10 min for membrane association, which is also a relatively brief duration. However, noteworthy differences emerged: while the PEI-25-mediated PIP3 complexes occasionally stained the nuclear perimeter within 18 min, the Q-starch/PIP3 complexes remained on the membrane for 4 h without entering the cells. It was not specified how the entry into the nucleus affected the animals and the activity of the cells over time.

### 2.4. Evaluating Exogenous PIP3 Biological Activity by AKT Reactivation

The cellular imaging studies (in Figure 2) showed PIP3 localization in the plasma membrane but did not prove the biological activity of PIP3. For example, to activate Akt, the phosphoinositol ring of PIP3 must be available for the binding of upstream Akt kinases (like PDK1). The next objective was to investigate whether the introduction of external PIP3 (both free and in the Q-starch/PIP3 complexes) could effectively preserve the biological functionality of PIP3 and trigger the AKT signaling pathway.

**Figure 3:**
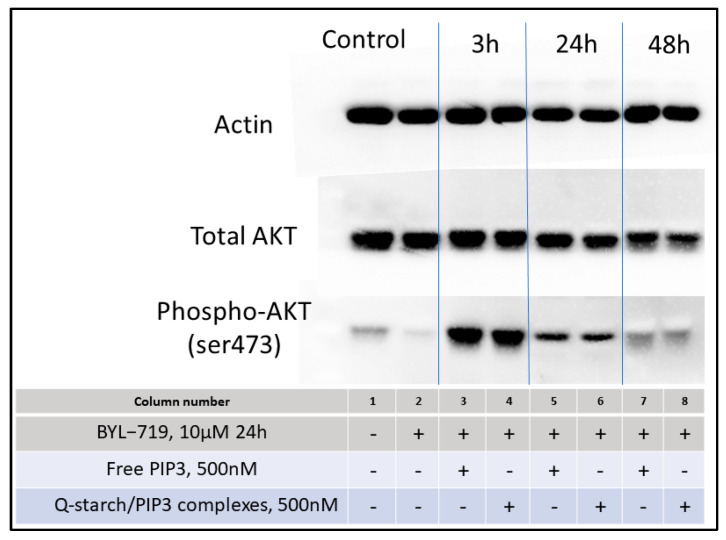
Western blot analysis panel displaying total protein lysates extracted from BJ fibroblast cells subjected to treatment with BYL-719, PIP3, or/and Qstarch/PIP3 complexes, as descried in the table below the panel (the ‘+’ sign signifies that the cells underwent that specific treatment). The panel shows the levels of actin, used as a loading control, along with total AKT and phospho-AKT (Ser473) expression, to evaluate the impact of free PIP3 and Q-starch/PIP3 complexes on AKT subsequent to PI3K inhibition by BYL-719, after different times (indicated at the top of the panel).

Western blot analysis was conducted to evaluate the impact of free PIP3 and the Q-starch/PIP3 complexes on the phosphorylation of AKT in BJ fibroblast cells. To ensure that Akt phosphorylation was the result of the biological activity of the administered PIP3 and was not reflective of endogenously generated PIP3, PI3K was inhibited by BYL-719 [52,53,54]. Subsequent to the inhibition, the cells were treated either with the Q-starch/PIP3 complexes or with free PIP3 for different times, i.e., 3, 24, and 48 h, to determine if reactivation of AKT was achieved. The attention was directed toward the phosphorylation site phospho-AKT Ser473 (pAKT), known to be essential for Akt activation [55,56]. Figure 3 presents a Western blot panel of pAKT activity in BJ fibroblast cells after PI3K inhibition and subsequent treatment with either free PIP3 or Q-starch/PIP3. Column 2, compared to column 1, demonstrates the effective inhibition of PI3K and endogenous PIP3 production, as reflected by the decreased pAkt signal. Cells treated with the Q-starch/PIP3 complexes or free PIP3 after BYL-719 inhibition (columns 3–8) presented activation at the pAKT site at all times tested.

The initial signal after 3 h (columns 3–4) reflects the activation of pAKT, gradually diminishing over time, as observed at 24 (columns 5–6) and 48 h (columns 7–8). There was no visible difference in pAKT signal intensity between treatments employing free PIP3 or the complexes. Comparable findings were observed in HaCAT cells, as presented previously [43].

PI3K is responsible for the endogenous generation of PIP3 by phosphorylating the 3’-hydroxyl group of PIP2. This process could be impaired in chronic wound healing [57,58]. Here we showed, by pharmacologically inhibiting PI3K and effectively inhibiting endogenous PIP3 production, that the extracellular delivery of PIP3 can circumvent potential defects in PI3K activation and endogenous PIP3 production.

Two pivotal conclusions arise from these findings. Initially, the observed biological activation of AKT by free PIP3 stands in contrast to the prior assumption that negatively charged PIP3, due to electrostatic repulsion with the cell membrane, would not penetrate cells. We assumed that the PIP3 structure, particularly, its hydrophobic characteristics, would facilitate its adhesion to the cell membrane. Consequently, the inositol head active sites manage to cross the membrane and activate intracellular processes. Equally significant is the determination that complexed PIP3 retained its biological activity. Notably, not only did it retain this activity, but the levels of activation observed via Western blot analysis were comparable to those achieved with free PIP3. From this, we can infer that the amount of PIP3 released from the complexes remained relatively consistent with that of free PIP3. Essentially, this suggests that the entirety of the complexed PIP3 (given that equal amounts were initially added) was released, hence generating a similar effect as free PIP3. Alternatively, a PIP3 quantity threshold may exist that would influence the duration of the PIP3 effect, implying that an excess amount beyond this threshold would not significantly alter the observed impact.

Further exam of the Western blot analysis indicated the activity window of PIP3, peaking within a brief period, after around 3 h, and gradually returning to the normal cellular levels after approximately 48 h. These findings have implications for the duration of future PIP3 treatments. In scenarios involving repeated treatments, consideration should be given to daily administration, ensuring intervals no longer than 48 h between doses.

### 2.5. Cytotoxicity Assay of Free PIP3 or Q-Starch/PIP3 Complexes In Vitro

AKT significantly influences apoptosis, cell metabolism, proliferation, and various cellular processes. In the Western blot assay, both samples treated with PIP3 and the Q-starch/PIP3 complexes exhibited heightened pAKT activity for approximately 48 h, which then returned to levels similar to those in the untreated control cells. To explore the impact of AKT activation on cell viability, we examined it on BJ fibroblast cells using crystal violet, 24 h after returning to the normal AKT levels, for a total posttreatment duration of 72 h.

**Figure 4:**
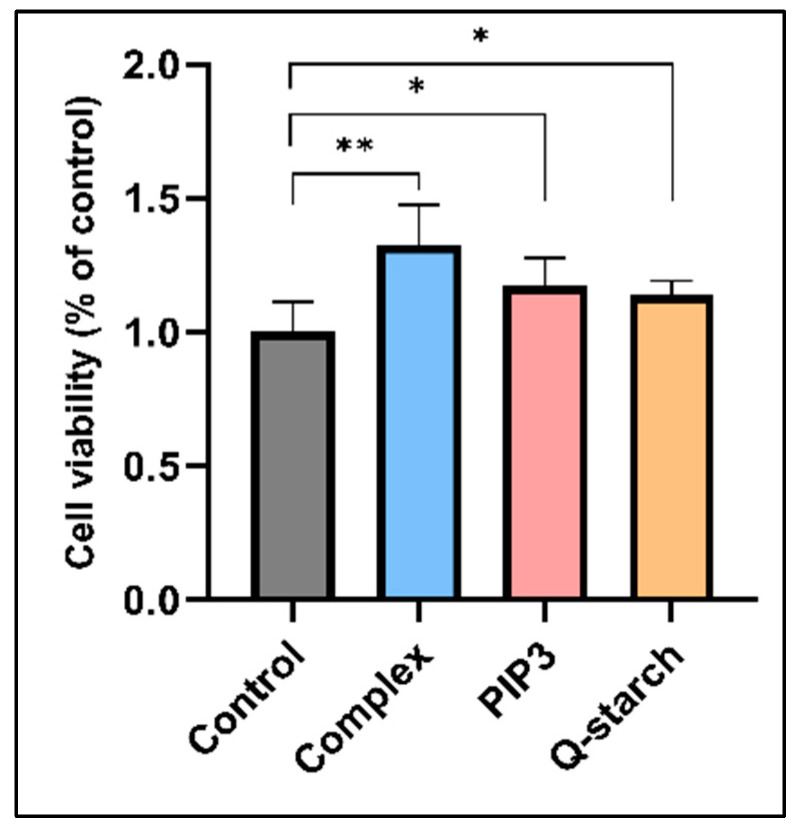
Results of the cell viability experiment conducted on fibroblasts subjected to the indicated treatments. Cell viability was measured after 72 h using crystal violet. The results are normalized to the those of the control group. Q-starch/PIP3 complexes at a N/P ratio of 2, PIP3 concentration of 500 nM, free Q-starch concentration corresponding to that in the complexes. (* *p* < 0.05, ** *p* < 0.01) Average + SEM (*n* = 6).

As depicted in Figure 4, all treated groups displayed heightened cell viability in comparison to the untreated control group. Particularly noteworthy was the significantly increased vitality observed in cells treated with the Q-starch/PIP3 complexes. These findings indicate the safety of both Q-starch/PIP3 complex and free PIP3 treatments for cells, suggesting their potential not only to maintain cell health but also to enhance cell proliferation.

While we did not directly measure the proliferation rates, the viability results strongly suggested increased cell numbers after 72 h, indicative of proliferation. The observed increase in proliferation corresponded directly to the Western blot results indicating AKT activation. Its involvement in anti-apoptotic processes is notable, as activated AKT suppresses apoptotic pathways by phosphorylating and deactivating pro-apoptotic proteins, ensuring cell survival, and allowing continuous cell division [57,58]. Moreover, AKT signaling stimulates protein synthesis via mTORC1 activation, facilitating the production of proteins essential for cell growth and proliferation [59,60]. Additionally, AKT regulates cellular metabolism, enhancing nutrient uptake and utilization, thereby providing the necessary resources for sustained cellular growth and division [61,62]. Furthermore, AKT involvement in amplifying growth factor signaling, triggered by factors like insulin-like growth factor 1 (IGF-1) or epidermal growth factor (EGF), initiates cascades of events that promote cell growth and division, contributing to increased proliferation [63,64]. 

In previous studies, exogenous PIPs were introduced into cells in vitro through methods involving acetoxymethyl esters, trademark carriers such as histones or neomycin, or polycationic carriers such as polyamines [65,66,67,68]. Studies showed that polyethylenimine is a suitable carrier; yet, its cellular toxicity prevents its application [69,70]. Alternative lipid transfection agents like vectamidine failed to facilitate the intracellular delivery of PIPn-NBD or Ins(1,4,5)P3-XRITC [71]. Polysaccharides such as Q-starch allow customization of size, shape, and surface properties. They offer precise control over chemical composition, interactions with drugs, and biological systems via tailored functional groups [72,73,74]. Moreover, polymeric carriers tend to be more stable and durable, providing robust platforms for drug delivery systems, including the sustained and targeted release of therapeutic compounds. The findings in this study indicate that Q-starch may serve as a safe carrier for PIP3, exhibiting no cytotoxicity and demonstrating membrane uptake abilities.

### 2.6. Collagen Production in BJ Fibroblasts Post Exogenous PIP3 Delivery

**Figure 5:**
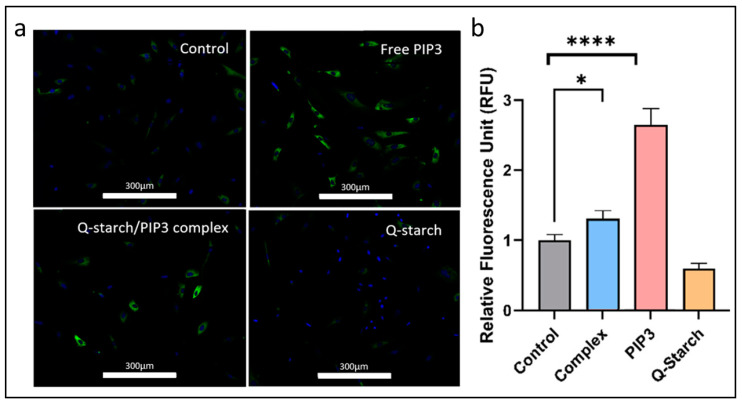
(**a**) Representative fluorescent images of BJ fibroblast cells 96 h post treatment. White lettering indicates the treatment. Bar—300 µm. Collagen type I, labeled in green, nucleus, labeled in blue. (**b**) Measurements of the fluorescence intensity relative to the cellular surface area in fibroblast cells subjected to the examined treatments, 96 h after treatment. Measurements were conducted using CellProfiler software, (* *p* < 0.05 and **** *p* < 0.0001).

Although not directly involved, PIP3 can exert an indirect influence on collagen synthesis through diverse pathways. Collagen, a critical protein in skin and connective tissues, is instrumental in the wound-healing process [75,76]. The process of collagen synthesis occurs mainly in fibroblasts. As healing progresses, fibroblasts migrate to the wound, producing collagen that forms the framework for tissue repair, with the initially deposited type III collagen eventually being replaced by type I collagen [76]. This remodeling strengthens the healed tissue and leads to scar formation. Collagen-based products are often used in wound care to promote healing and maintain an optimal wound environment [77]. Fibroblasts are the main cells in connective tissue responsible for producing the extracellular matrix, with collagen being their most abundant output [78]. In this study, we investigated the influence of free PIP3 and the Q-starch/PIP3 complexes on collagen synthesis in BJ fibroblast cells. The cells underwent treatment with either PIP3 or the Q-starch/PIP3 complexes at a concentration of 500 nM. Subsequent to a 96-h incubation period at 37.0 °C under a 5% CO_2_ atmosphere, type I collagen presence was ascertained via immunofluorescence techniques. Figure 5a presents fluorescence microscopy images describing the four experimental conditions: control, i.e., untreated cells, cells treated with free PIP3, cells treated with the Q-starch/PIP3 complexes, and cells treated with the free Q-starch carrier. Quantitative image analysis, in Figure 5b, was executed using CellProfiler software v4.2.6, assessing the fluorescence intensity relative to the cellular surface area [64]. The subsequent graphical representation depicts the fluorescence intensity normalized against that of the untreated control group.

It is evident from the data that both free PIP3 and its complexed form considerably augmented collagen synthesis, emphasizing PIP3 pivotal role in modulating collagen-related intracellular pathways along with AKT-related processes. AKT signaling can intersect with pathways like that activated by TGF-β, which directly regulates collagen production [79,80]. AKT involvement in cell survival and growth can indirectly impact the processes necessary for collagen synthesis and deposition within the extracellular matrix (ECM). Moreover, AKT can also affect the expression of enzymes like matrix metalloproteinases and tissue inhibitors of metalloproteinases, which play crucial roles in collagen turnover and remodeling. By influencing these enzymes, AKT indirectly participates in the regulation of the collagen levels within tissues.

Collagen is the core protein in the ECM, where collagen fibers establish a scaffold bolstering cells, tissues, and organs, fortifying their resilience and cohesion [81,82,83]. However, an abundance of neutrophils in chronic wounds triggers an over-production of reactive oxygen species (ROS), resulting in direct ECM damage [84]. Moreover, neutrophils release serine proteases like collagenase (MMP-8), which deactivate ECM elements [85,86]. This ECM degradation impedes cell migration, diminishes fibroblast proliferation, and hampers collagen synthesis [87,88,89]. Furthermore, the breakdown products of the ECM foster inflammation and a perpetuating cycle of self-sustaining inflammation.

Enhancing collagen quantity and organization using various agents has been directly linked to expediting and enhancing the wound healing process [90,91,92,93,94]. Research indicates a clear correlation between the application of PDGF, an approved biological therapy for chronic wounds, and elevated collagen production [95]. Hence, treatment involving PIP3 offers the potential for comparable collagen-related outcomes in chronic wound care.

As PIP3 has shown its capacity to initiate mechanisms linked to collagen production, it holds potential for addressing conditions involving reduced collagen levels, such as aging-related changes or wrinkle formation [96,97,98].

### 2.7. Evaluating Cell Migration Post Exogenous PIP3 Delivery by the Scratch Assay

**Figure 6:**
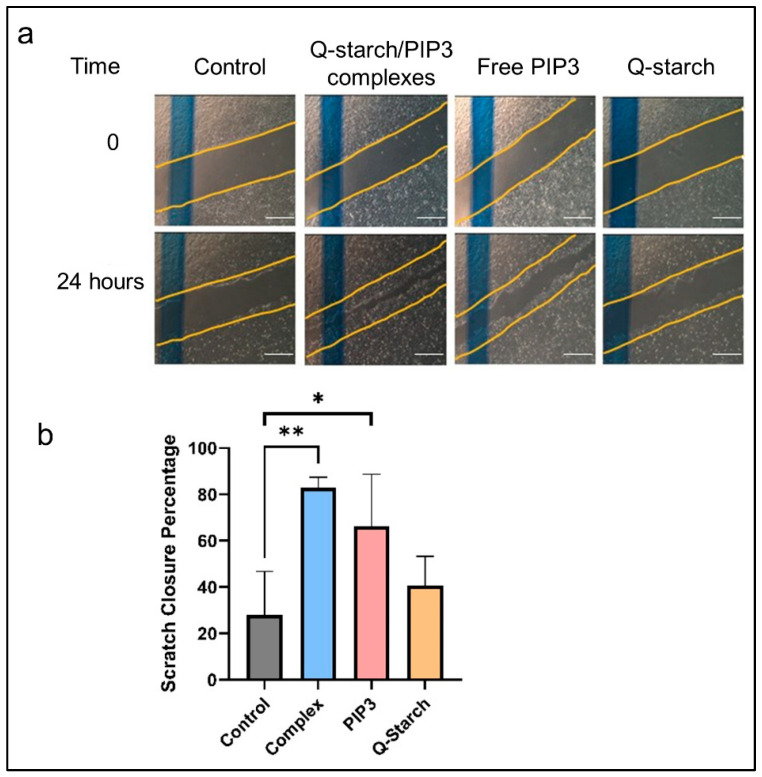
(**a**) Representative images of the scratch assay conducted on HaCAT cells subjected to the indicated treatments. The top row shows images at time 0, immediately after performing the scratch, and the bottom row shows images captured 24 h posttreatment. Blue marking is for photographic purposes only. Yellow dragline was added in the figures to follow the progress of the cell migration in relation to time 0. Q-starch/PIP3 complexes at a N/P ratio of 2, PIP3 concentration of 500 nM, free Q-starch concentration corresponding to that in the complexes. Average + SEM (*n* = 3). Bar—750 µm. (**b**) Results of a scratch assay experiment that was conducted on HaCAT cells subjected to the indicated treatments. The scratch closure measurement was performed after 24 h and compared to the measurement at time 0. (* *p* < 0.05, and ** *p* < 0.01).

After observing increased collagen production upon the introduction of PIP3 and its complexes, the next objective was to investigate whether the addition of PIP3 can also potentially accelerate cell migration, one of the main features of wound closure. For this, a scratch assay using HaCAT cells was conducted. This assay quantifies the rate at which cells migrate to occlude a designated gap preformed in the middle of a layer of fully confluent cells. Photographic documentation was procured immediately subsequent to the creation of the scratch (denoted as time t = 0) and following a 24 h incubation period with the Q-starch/PIP3 complexes, free PIP3, or free Q-starch (Figure 6a). The migration of the cells towards the closure of the scratch after 24 h can be seen. The area of each scratch was measured using ImageJ and calculated relative to time 0, as presented in Figure 6b. The cells subjected to the treatment with the Q-starch/PIP3 complexes presented the highest closure percentage. The cells treated with free PIP3 also presented accelerated migration for gap closure relative to the untreated control cells. For the cells treated with free Q-starch, a slight increase in migration was observed in comparison to the control group, although this increase lacked statistical significance.

The faster scratch gap closure in response to PIP3 and the Q-starch/PIP3 complexes can be explained by the effect of AKT on cell migration. AKT orchestrates cytoskeletal dynamics, promoting the formation of protrusions like lamellipodia and filopodia, essential for cellular movement. AKT influence extends to cell adhesion and detachment, regulating the intricate balance necessary for cells to attach to and detach from surfaces during migration. Additionally, AKT signaling modulates various molecules and pathways involved in migration, including integrins and focal adhesion kinase (FAK), steering the directionality and speed of cellular movement. Its involvement downstream of growth factor receptors, like insulin-like growth factor 1 (IGF-1) or epidermal growth factor (EGF), initiates cascades that alter cytoskeletal organization and motility, pivotal for cell migration.

### 2.8. Examination of Wound Healing following Exogenous PIP3 Delivery

Following the evaluation of PIP3 and the Q-starch/PIP3 complexes at the cellular level in vitro, the next step was assessing their therapeutic efficacy in vivo in a murine dorsal wound model [99,100,101]. Two distinct wound healing assays were performed, involving (1) an incisional wound model subjected to daily injection into the wound margins followed by 12 days of observation; (2) an excisional wound model subjected to a singular treatment by injection into the wound margins and a subsequent 5-day observation period. In both experiments, the examined groups were control (with incisional/excisional wound, without treatment, i.e., subjected to saline injections), free PIP3, Q-starch/PIP3 complexes, and free Q-starch. After wounding, C57BL/6 mice were treated with injections of 40 µL of the tested materials in four sites of the wound margins.

**Figure 7:**
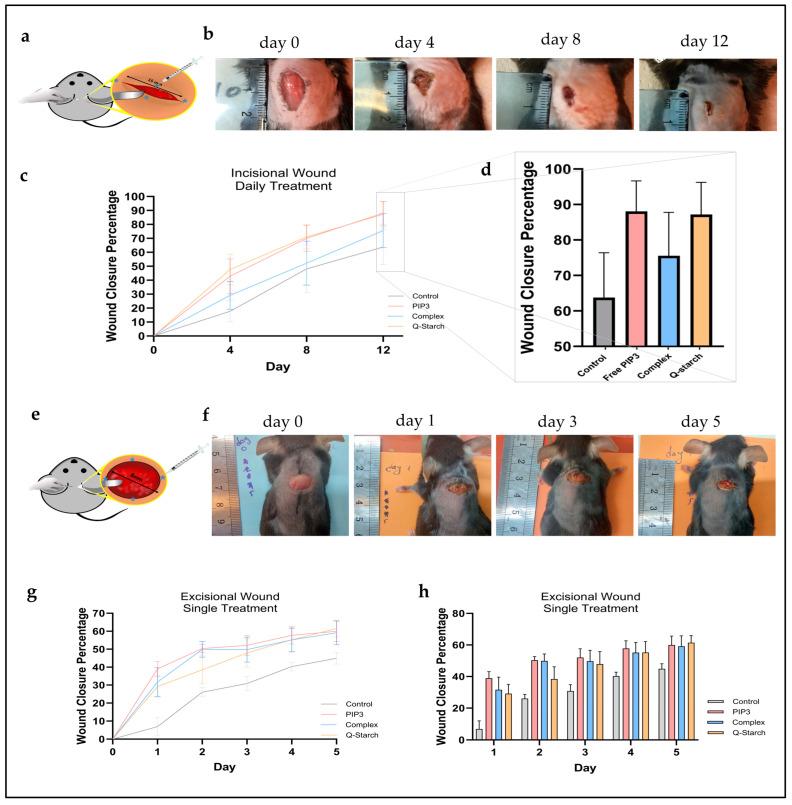
Analysis of the wound closure percentages in a wound healing assay encompassing four distinct treatment groups: control, PIP3, 500 nM, Q-starch/PIP3 complexes, PIP3, 500 nM, N/P ratio of 2, and free Q-starch at a concentration corresponding to that in the complexes. (**a**) Incisional wound on the mouse back, with a length of 15 mm. The wound was treated by injection into 4 sites in the wound margins, marked with a blue star. (**b**) Representative images of incisional wound healing in a mouse treated with 40 µL of free PIP3 at a concentration of 500 nM. Photographs were taken every four days. (**c**) Analysis of incisional wound healing in mice treated by injection of 40 µL of the examined substances into the wound margins. The analysis focused on quantifying the percentage of wound closure relative to the initial wound size on day 0. Average + SEM (*n* = 4). (**d**) Wound closure percentage on the 12th day of the experiment. (**e**) Excisional wound made on the mouse back with an 8 mm biopsy punch. The wound was treated by injection into 4 sites of the wound margins, marked with a blue star. (**f**) Representative images of excisional wound healing in a mouse treated with 40 µL of free PIP3 at a concentration of 500 nM. Photographs were taken on wounding day (day 0), and on day 1, 3, and 5 after wounding. (**g**) Analysis of excisional wound healing in mice treated by injection of 40 µL of the examined substances into the wound margins. Analysis focused on quantifying the percentage of wound closure relative to the initial wound size on day 0. Average + SEM (*n* = 3). (**h**) A bar graph extracted from (**g**), included for a convenient comparison of the treatments on each day.

Figure 7a shows an illustration of the incisional model, wherein a 15 mm cut was created using a scalpel, and the locations of the treatment injections are denoted by a star symbol. Figure 7b presents visual representations of the stages of wound healing in a mouse that received treatment with free PIP3 at a concentration of 500 nM. Figure 7c presents the analysis of the incisional wound closure percentage relative to the wound size on the day of injury through measurements on day 4, 8, and 12. In Figure 7d, the depicted data show the extent of wound closure achieved on the 12th day of the experimental study. Notably, wounds subjected to PIP3 and the Q-starch/PIP3 complexes exhibited accelerated wound closure rates by about 12–25% in comparison to the control group. Furthermore, wounds treated with free Q-starch also demonstrated expedited wound closure relative to the control group. The accelerated closure rates observed with free PIP3 and the complexes were relatively similar, within error, and were attributed to equivalent PIP3 concentrations available in both treatments, of 500 nM. Despite being enclosed within a Q-starch/PIP3 complex, PIP3 retained its biological activity in vivo. Regarding Q-starch, the accelerated healing is assumed to be associated with the anti-bacterial and anti-inflammatory properties inherent to the quaternary amine [102,103].

A singular-administration assessment was conducted to discern the potential sustained effects of PIP3 when complexed. Figure 7e illustrates an excisional wound model created on the mouse back using an 8 mm biopsy punch. A single treatment was administered by injecting each examined substance into the wound margins, as indicated by a star. Figure 7f presents representative images depicting the stages of wound healing in a mouse treated once with a free PIP3 solution at a concentration of 500 nanomolar (nM), followed up for a period of 5 days. The analysis of the excisional wound closure percentage relative to the day of injury from day 1 to 5 is presented in Figure 7g,h. Figure 7h, a bar graph extracted from Figure 7g, was included for a convenient comparison of the treatments on each day. These data validated earlier findings, with wounds treated with free PIP3 or its complexes demonstrating higher closure rates compared to the controls.

From the single treatment model, it was observed that, over the course of the 5-day wound closure, the closure percentages observed in both the free PIP3-treated and the complex-treated groups exhibited similarity. This alignment supports the presumption that there exists a specific concentration threshold of PIP3, beyond which additional quantities cease to contribute to AKT activity.

All treatment modalities, excisional and incisional, facilitated wound closure without injection-site erythema or hypersensitivity, wound necrosis, or detectable alterations in murine behavior and weight metrics. This affirms that, within the employed concentrations, both free PIP3 and its complexed form retained a safe profile for dermal administration. This finding encourages further exploration of treatments with PIP3 or Q-starch/PIP3 complexes for chronic wounds.

## 3. Materials and Methods

### 3.1. Starch Quaternization

Soluble hydrolyzed potato starch (Mw 26,765 Da) was dissolved in a sodium hydroxide solution (0.19 g/mL) to achieve a starch concentration of 50 mg/mL. This mixture was stirred (100 rpm, AGE magnetic stirrer, Velp Scientifica) for 40 min at room temperature. The quaternization agent, a 3-chloro-2-hydroxypropyltrimethyl-ammonium chloride (CHPTAC) solution, was diluted with double-distilled water (DDW, 18.3 MΩ·cm) to reach a concentration of 0.32 g/mL before its gradual introduction into the starch blend. The entire mixture was then stirred for a full day in ambient conditions. To precipitate the resulting compound, a mixture of ethanol and acetone (acidified with 1% vol. HCl, in a 1:3% vol. ratio) was combined with the starch solution in a 4:1 ratio. The product was rinsed with 80% vol. ethanol four times, redissolved in 10 mL of DDW, and transferred into a 14 KDa molecular weight cut-off dialysis tube. This tube was dialyzed in 5 L of DDW for two days, refreshing the water two times. Ultimately, the resultant solution underwent freeze-drying and lyophilization for three days, yielding the refined quaternized starch (Q-starch).

### 3.2. Quaternized Starch Labeling

Q-starch molecules were tagged with 5-(4,6-dichlorotriazinyl) amino fluorescein (5-DTAF) for visualization by fluorescent microscopy. A solution was prepared by dissolving 100 mg of Q-starch in 3 mL DDW and adjusting its pH to 11–12 using 1 M NaOH. This solution was agitated (100 rpm, AGE magnetic stirrer, Velp Scientifica) at ambient temperature for half an hour. A dissolved 5-DTAF solution (7.5 mg in 0.3 mL of DMSO) was introduced into the Q-starch blend, which was then stirred at room temperature for 24 h while shielded from light. Post stirring, it was neutralized using 0.1 M HCl and transferred into a 14 KDa molecular weight cut-off dialysis tube. This tube was submerged in a PBS solution (pH 7.4) for three days, followed by two days in DDW (18.3 MΩ·cm). The final labeled Q-starch^DTAF^ product was procured after freeze-drying and lyophilizing the dialyzed solution for three days to ensure total dryness.

### 3.3. Q-Starch/PIP3 Complex Preparation

PIP3 used in the delivery experiments was identified as D-myo-phosphatidylinositol 3,4,5-trisphosphate (Ptdlns(3,4,5)P3, P-3916, Echelon Bioscience, Salt Lake City, UT, USA). Complexes were formed by adding the Q-starch solution (0.4 mg/mL) to a vial containing a PIP3 solution (0.6 mg/mL) that had been pre-sonicated for 60 s. Both solutions were dissolved in DDW. Complex formation was guided by the N/P molar ratio, where ‘N’ signifies the molar portion of positive nitrogen groups on the carrier, and ‘P’ represents the molar portion of negative phosphate groups on PIP3. The Q-starch/PIP3 quantity for a particular N/P ratio was determined by the nitrogen content (%N by weight). For example, for obtaining Q-starch/PIP3 complexes at a N/P ratio of 2, 4.4 µL (0.4 µg/µL) of Q-starch solution was added to tubes holding 1 µL (0.6 mg/mL) of PIP3 solution that had been pre-sonicated for 60 s. For the in vitro intracellular assays with fluorescence labeling, Q-starch^5-DTAF^-based carrier complexes were employed. Adhering to a N/P ratio of 2, 1.85 µL (0.4 µg/µL) of Q-starch^5-DTAF^ (Ex/Em—495/515) was added to tubes with 0.5 µL (0.6 mg/mL) of the PIP3 solution pre-sonicated for 60 s. After brief vortexing, the solutions at varying N/P ratios were allowed to settle at room temperature for 40 min to facilitate complex formation. While the PIP3 quantity remained consistent, the added carrier amount was adjusted to achieve the target N/P ratio.

### 3.4. Q-Starch/PIP3 Complex Characterization

#### 3.4.1. Q-Starch/PIP3 Complex Size Measurements Using DLS

The hydrodynamic radius of the complexes underwent measurement using DLS. The Q-starch/PIP3 complexes were prepared following the previously described method, achieving concentrations of 250 nM PIP3 in Eppendorf tubes, reaching a final volume of 1 mL. Spectra were captured utilizing a CGS-3 goniometer from ALV (Langen, Germany). The laser power was set at 20 mW, operating at the He–Ne laser line (632.8 nm). Correlograms were computed via an ALV/LSE 5003 cross-correlator (Langen, Germany) positioned at 90 degrees, for 10 s over 30 iterations, at a temperature of 25 degrees Celsius. Each sample was enclosed in a thin-walled cylindrical glass cuvette and submerged in toluene, serving as the optical matching fluid, within a container. Subsequently, measurements were taken for each complex sample.

#### 3.4.2. Q-Starch/PIP3 Complex Structural Characterization Using Cryo-TEM

The morphology of the complexes, as well as a confirmation of their size, was evaluated by cryo-TEM. The Q-starch/PIP3 complexes were prepared to reach a final concentration of 250 nM PIP3 in Eppendorf tubes in a final volume of 1 mL. Specimens of Q-starch/cargo complexes were prepared on a copper grid coated with a perforated lacey carbon film, 300 mesh (Ted Pella Inc., Redding, CA, USA), under controlled temperature (cryogenic temperature). A 3 µL drop of the analyzed solution was applied to the grid and blotted with filter paper to form a thin liquid layer. The blotted samples were immediately and automatically plunged into liquid ethane at its freezing point (−183 °C) using Plunger (Leica EM GP, Wetzlar, Germany). The specimens were transferred into liquid nitrogen for storage. Samples were analyzed using FEI Tecnai 12 G2 TEM at 120 kV with a Gatan cryo-holder maintained at −180 °C. Images were recorded on a slow-scan cooled charge-coupled device camera (Gatan, Pleasanton, CA, USA) in low-dose conditions to minimize electron-beam radiation damage. The recording was carried out using the Digital Micrograph GMS3 software package.

#### 3.4.3. Q-Starch/PIP3 Complex Size and Structural Characterization Using AFM

Wet atomic force microscopy was utilized to visualize the size and morphology of the Q-starch/PIP3 complexes. Seven microliters of the Q-starch/PIP3 complexes was dispensed onto individual freshly cleaved mica sheets. The complexes were incubated at room temperature for 5 min. The AFM measurements were carried out using a Cypher-ES system (Asylum Research/Oxford Instruments, Santa Barbara, CA, USA) in AC mode, employing an AC 160 probe.

#### 3.4.4. Q-Starch/PIP3 Complex Zeta Potential Measurements

The zeta potential, representing the surface charge of each complex, was assessed using a zeta sizer. Q-starch/PIP3 complexes were prepared following the outlined procedure, achieving a concentration of 50 nM PIP3 within Eppendorf tubes, reaching a final volume of 1 mL. Subsequently, the samples were transferred to a U-tube cuvette (DTS1070C, Malvern, UK) for zeta potential measurements employing the Zetasizer (ZNNanoSizer, Malvern, UK). The assessment encompassed samples containing free PIP3, free Q-starch, and complexes at varying N/P ratios, ranging from 1 to 3, and was conducted in an automated mode at 25 °C. The Smoluchowski model served as the basis for zeta potential calculations.

### 3.5. Cell Culture Handling

BJ fibroblast and HaCAT cells were chosen for the in vitro examinations to investigate the cellular uptake and the biological activity of the complexes in the cells. The cells were cultivated in DMEM growth medium, which included 4.5 mM glucose, 10% fetal bovine serum (FBS), 1% L-glutamine (2 mM), and 1% penicillin–streptomycin (at concentrations of 100 µg/mL each). Cell growth took place in flasks with a capacity of 75 cm^2^. Every 3–4 days, the cells were divided using 2–3 mL of trypsin–EDTA and distributed among 3–4 flasks to avoid excessive cell confluence and over-crowding. After splitting, the trypsin’s effects were countered with 10 mL of growth medium. Proper pipetting ensured a uniform cell suspension, and the cells were then counted with the Countess™ II FL (Invitrogen, Carlsbad, CA, USA) Automated Cell Counter. Counting was performed by mixing 20 μL of each cellular suspension with 20 μL of a 0.4% trypan blue solution (in a 1:1 *v*/*v* ratio). All processes related to cell culture were executed in a sanitized laminar-flow hood, which was cleaned with UV light and 70% ethanol prior to each use.

### 3.6. Cellular Uptake of the Q-Starch/PIP3 Complexes

For the cellular uptake analysis, live confocal microscopy was employed. In total, 5 · 10^4^ BJ fibroblast or HaCAT cells/well were seeded in an eight-well Ibidi µ-Slide plate with a glass bottom (Cat. No 80827) and allowed to adhere for 24 h. Prior to treatment, the cells underwent two PBS washes and were subsequently stained using one or multiple dyes, as recommended by their respective manufacturers. NucBlue was used for nucleus visualization (Ex. 405 nm, from Invitrogen, Carlsbad, CA, USA). Post-staining, another double PBS wash was executed before introducing 300 µL of the selected complexes to achieve a final PIP3 concentration of 500 nM. A 4 h incubation at 37 °C in a 5% CO_2_ environment followed. Subsequently, for HaCAT cells (concentration and time determination), the Perkin Elmer’s spinning disc system Ultra View ERS Rapid Confocal Imager (Waltham, MA, USA) was used for image capture. For BJ cells, Zeiss LSM 880 (Oberkochen, Germany) was used with the Plan-Apochromat 10×/0.45 lens. To monitor the intracellular uptake of the Q-starch/PIP3 complexes microscopically, the Q-starch was fluorescent labeled. For the complexes, fluorescence from the labeled Q-starch^DTAF^ (excited at 488 nm) was observed.

### 3.7. Cell Viability/Cytotoxicity

In the cell viability/cytotoxicity assay employing crystal violet staining, the primary objective was to assess the impact of the Q-starch/PIP3 on the viability of cultured cells. BJ fibroblast cells were initially seeded in 24-well plates and permitted to adhere overnight under standard culture conditions. Subsequently, the culture medium was replaced with fresh medium containing the test substance, i.e., free PIP3, Q-starch/PIP3 complexes, and free Q-starch; a control group receiving no treatment was also assessed. After a designated incubation period of 72 h, the cells were fixed with 4% paraformaldehyde in phosphate-buffered saline (PBS) for 20 min at room temperature. Following fixation, excess paraformaldehyde was removed, and the cells were gently rinsed with distilled water. Crystal violet staining was then performed using a 0.1% crystal violet solution, allowing for incubation for 20 min at room temperature. After staining, excess dye was washed using PBS, and the plates were allowed to air-dry for 24 h. The solubilization of the crystal violet dye was achieved by adding 200 µL of acetic acid 10% to each well, followed by gentle mixing. Subsequently, the absorbance of the samples was measured at 570 nm using a microplate reader. Statistical analysis was employed to assess significant differences between the treatment groups using Prism software v10.1.1.

### 3.8. Scratch Assay

HaCAT cells were cultured, processed, and treated directly in 12-well plates using the growth medium. The cells were incubated for 24 h at 37 °C until they achieved full confluence. Then, the cells were subjected to 12 h of starvation conditions using DMEM without FBS. Afterward, the growth medium was discarded, and the cells were rinsed with PBS twice. A scratch was created in the cell monolayer using a p200 pipet tip. To refine the scratch boundaries, the cells were washed with DMEM. The cells that remained untreated served as a control. After the scratching procedure, freshly prepared complexes with a N/P ratio of 2 and a free PIP3 concentration of 500 nM in a total volume of 1 mL were added to the wells. The 12-well plates were then incubated at 37 °C for a cumulative duration of 36 h. Observations and imaging were conducted 24 h posttreatment using an Olympus BX53 microscope. Analysis was performed by calculating the scratch percentage closure using ImageJ software v1.53k. Statistical analysis to assess significant differences between the treatment groups was conducted using Prism software v10.1.1.

### 3.9. AKT Activation by Free PIP3 or the Q-Starch/PIP3 Complexes Assessed by Western Blot

This experiment was conducted following a method similar to that previously described [43]. BJ cells were cultured at a starting density of 5 · 10^5^ cells/mL in 60 mm dishes, at 37 °C and in 5% CO_2_ in DMEM. Over 24 h, the cells achieved approximately 80% confluence. Four treatment groups were examined: firstly, a control set was left untreated and incubated for an additional 24 h; secondly, a group underwent treatment with 10 µM PI3K inhibitor (BYL719) and incubation for 24 h; a third set was initially treated similarly to the second one but subsequently exposed to 500 nM free PIP3 for a duration of 3, 24, and 48 h; and lastly, the fourth group was subjected to a treatment w 500 nM Q-starch/PIP3 complexes for 3, 24, and 48 h after a 24 h PI3K inhibitor incubation. Posttreatment, the cells in the dish were cooled on ice and subsequently rinsed twice with ice-cold PBS. After removing PBS, a lysis buffer was added to the cells. Utilizing a plastic scraper, the adherent cells were detached and relocated to microcentrifuge tubes. A centrifugation at 4 °C for 10 min at 14,000 rpm was executed, after which the tubes were carefully taken out and placed on ice. The supernatant was shifted to a fresh, ice-cold tube. Protein concentrations in the samples were discerned using the Bradford assay. A volume of 20 µL of each sample was pipetted into 96-well plates, combined with 180 µL of diluted Bradford reagent (1:5 in DDW). These plates were analyzed using the Tecan Infinite M200 plate reader at 595 nm. Depending on the concentrations determined, the samples were diluted and subsequently heated to 95 °C for 5 min. Equivalent protein volumes (totaling 20 µg) were electrophoresed on SDS-PAGE gels. Post-electrophoresis, the gels were sandwiched between transfer membranes and subjected to the blotting procedure following the manufacturer’s protocol. The membranes were incubated in a blocking solution and agitated gently for an hour at ambient temperature. Primary antibodies targeting actin, P-AKT 473, and total AKT were added at a dilution of 1:1000 and left overnight in a refrigerated setting. All antibodies were purchased from Cell Signaling. After a series of washes with TBST, secondary anti-rabbit antibodies conjugated to horseradish peroxidase were introduced for an hour. After the incubation period, the membranes were immersed in an enhanced chemiluminescence solution and imaged using the c300 Azure camera system.

### 3.10. Collagen Production in BJ Fibroblasts

BJ fibroblast cells were cultured on an 18 mm round glass coverslip placed in 12-well plates until they reached approximately 60–70% confluence. In this experiment, four groups were established: a control group that received no treatment, a set of cells exposed to PIP3 at a concentration of 500 nM, another group treated with the Q-starch/PIP3 complexes at a N/P ratio of 2 and a PIP3 concentration of 500 nM, and finally, a group treated with starch at a concentration equivalent to that of the complexes. The cells were incubated for 96 h at 37 °C and in 5% CO_2_. The growth medium was then aspirated, and the cells were washed gently with PBS. The cells were fixed with 4% paraformaldehyde for 20 min at room temperature. After fixation, PFA was removed, and the cells were subjected to three washes with PBS. Cell permeabilization was achieved by treating the cells with 0.1% Triton X-100 in PBS for 10 min and placing them on ice. Following permeabilization, the cells were again washed three times with PBS. To block non-specific antibody binding, the cells were incubated in a blocking solution composed of 5% bovine serum albumin (BSA) in PBS for 1 h at room temperature. Post-blocking, the cells were treated with the primary antibody, goat anti-type 1 collagen, AF488 (Enco, Petach Tikvah, Israel), appropriately diluted in the blocking solution according to the manufacturer’s instructions. The plate was then placed on a shaker, gently shaken, and left for one hour at room temperature. Right after, the cells were washed twice with PBS to eliminate unbound primary antibodies. This was followed by mounting the cells with DAPI-Fluoromount (Southern biotech, Birmingham, AL, USA). A coverslip was placed over each round glass coverslip, ensuring no air bubbles were trapped. Fluorescence imaging was conducted using a fluorescence microscope, utilizing the appropriate filters for AF488 to observe collagen and DAPI for nuclei visualization. Quantitative image analysis was executed using CellProfiler software v4.2.6, assessing the fluorescence intensity relative to the cellular surface area. Statistical analysis to assess significant differences between treatment groups was performed using Prism software v10.1.1.

### 3.11. Wound Healing Assay

These experiments were performed in C57BL wild-type mice under the approval of the Council responsible for animal experiments in the State of Israel. Seventy-two hours before the wound was performed, the mice were treated with dipyrone in drinking water to relieve their pain. In the excisional wound assay, adult mice were anesthetized using a combination of ketamine and xylazine, ensuring their complete immobility during the procedure. Once sedated, their dorsal fur was trimmed, and the exposed skin was thoroughly disinfected with 70% ethanol to maintain sterility. Using an 8 mm biopsy punch, a full-thickness excisional wound was created on the dorsal skin. In the incisional wound healing experiments, a 15 mm incision was made using a scalpel. Subsequently, the mice were divided into four distinct treatment groups: (1) a control set that was not subjected to any treatment; (2) a group treated with 500 nM free PIP3; (3) another group that received 500 nM Q-starch/PIP3 complexes at a N/P ratio of 2; (4) a group that was injected with Q-starch at a concentration analogous to that in the complexes. The treatment, dispensed in a 40 µL volume, was precisely injected into the wound margins in 4 points, both in the excisional and in the incisional wounds. In the incisional wound healing experiments, daily treatment was performed, and in the excisional trial, a single treatment was performed on the day the wounds were made. Throughout the subsequent days, wound closure was monitored and documented via periodic photographs, providing insights into the effectiveness of each treatment approach. Pictures were taken using a SAMSUNG A72 cell phone camera in a macro mode from a fixed height of about 8 cm, with a ruler placed next to the mouse to later measure the area of the wound. Statistical analysis to assess significant differences between the treatment groups was performed using Prism software v10.1.1.

## 4. Conclusions

In this research, we assessed the influence of PIP3 and Q-starch/PIP3 complexes on keratinocyte and fibroblast cells, constituting over 95% of the skin cells and orchestrating wound healing dynamics through the AKT/mTOR pathway. The results of this study provide valuable insights into the potential therapeutic applications of PIP3 and its complexes with Q-starch in wound healing processes.

The Q-starch/PIP3 complex and free PIP3 treatments were found to be safe and did not affect cell viability; in fact, they appeared to enhance cell proliferation, an important feature in wound healing. Q-starch/PIP3 complexes are suitable in terms of size and zeta potential characteristics for cellular entry, accumulating within the cell membrane, without evidence of cytoplasmic infiltration, suggesting their potential for targeted action on the cellular membrane.

Treating fibroblast cells with PIP3 or the Q-starch/PIP3 complexes promoted heightened collagen production and accelerated cell migration, facilitating scratch closure in cell cultures. Furthermore, both free PIP3 and the Q-starch/PIP3 complexes effectively reactivated p-AKT activity following its inhibition. This reinstatement of AKT activity by these treatments is particularly significant in conditions like chronic wounds characterized by reduced AKT function. Moreover, in wound healing models in mice, both free and complexed PIP3 in both single and recurrent administration demonstrated a marked acceleration of wound closure compared to the control groups.

The finding that PIP3 can be complexed using Q-starch and preserve its biological activity suggests the possible application of the complexes in cases posing challenges related to PIP3 stability or its integration into a controlled release system.

Overall, these findings suggest that free PIP3 and the Q-starch/PIP3 complexes hold promise as therapeutic agents for chronic wound healing and cellular processes, offering potential applications in promoting tissue repair and inflammation management. It would be prudent to explore the application of PIP3 or the Q-starch/PIP3 complexes in chronic wound scenarios, such as those observed in patients with diabetes or other ulcers. The utilization of PIP3 in its various forms may stimulate the intracellular mechanisms imperative for wound resolution, preventing limb amputation and even saving many lives.

## Data Availability

The data presented in this study are available on request from the corresponding author.
